# Typical leiomyoma of the scrotum: A rare case report

**DOI:** 10.1016/j.ijscr.2020.01.057

**Published:** 2020-02-06

**Authors:** Saman Salih Fakhralddin, Rawa Bapir, Muhamed Hussen Babarasul, Zheen Bahaddin Ibrahim, Ismaeel Aghaways

**Affiliations:** a Sulaymaniyah Surgical Teaching Hospital, Urology Department, Iraq; b Shaheed Shawkat Haji Musheer Hospital/Said Sadiq, Sulaymaniyah, Iraq; c U-merge Ltd., (Urology in Emerging Countries), London, United Kingdom^1^; d Sulaymaniyah Surgical Teaching Hospital, Iraq; e University of Sulaymaniyah, Faculty of Medical Sciences, School of Medicine, Department of Surgery, Iraq

**Keywords:** Case report, Dartos muscle, Genital leiomyoma, Leiomyoma, Scrotum

## Abstract

**Introduction:**

Scrotal leiomyomas are benign tumors arising from the dartos layer of the scrotal wall. The exact cause of this tumor is not yet clear. Malignant transformation to leiomyosarcomas has been reported.

**Case presentation:**

A 52-year-old male who complained of a painless lump on the right side of the scrotum that lasted 12 years. Physical examination revealed a single, firm, and non-tender, mobile lump on the anterior aspect of the right scrotum. Both testes were normal and no inguinal lymph nodes could be palpated. Ultrasound scan of the scrotum showed a 40 mm × 20 mm hypoechoic, poorly vascular lesion in the scrotum. Under spinal anesthesia, the mass has been excised. Histopathological and immunohistochemical findings were consistent with the diagnosis of scrotal leiomyoma.

**Discussion:**

Leiomyomas may originate from any location in the genitourinary system where there is smooth muscle. Scrotal smooth muscle tumors can be categorized into Leiomyomas, atypical or symplastic leiomyomas, which are not hypercellular and lack mitotic activity, and leiomyosarcomas. Ultrasound is the first-line imaging investigation in patients with suspected scrotal masses. Typical leiomyomas and atypical ones behave similarly. Therefore, they are managed only with surgical excision, while leiomyosarcomas need a wide 3−5 cm margin resection. Both recurrence and malignancy have been described. Close follow-up is required to detect recurrence.

**Conclusion:**

Scrotal leiomyoma is a rare benign mesenchymal tumor of the middle-aged men. The current report describes the clinical and histopathological characteristics to help reduce erroneous diagnoses of this rare tumor.

## Highlights

• Scrotal leiomyomas are benign tumors arising from the dartos layer of the scrotal wall.
• It is the disease of the middle aged men.
• Malignant transformation to leiomyosarcoma has been reported.
• Surgical excision is the main line of management.

## 1 Introduction

Leiomyoma is a benign tumor of smooth muscle that can form nearly anywhere in the body. The uterus is known to be the commonest site of the tumor but scrotal leiomyoma is essentially a rare benign pathology arising from the dartos muscle [1]. Scrotal smooth muscle tumors are categorized to conventional or typical leiomyoma, atypical or symplastic leiomyoma that have bizarre nuclei may mimic malignancy, and leiomyosarcoma [2,3]. A multi-national review by Adebayo et al. conducted in 2018 found 94 cases of scrotal leiomyoma in the literature [4]. In line with SCARE criteria, we present a case with a single, solid scrotal leiomyoma in a 52-year-old man [5].

## 2 Case presentation

A 52-year-old man presented with a painless mass in the right side of the scrotum for 12 years. There was no history of trauma, infection, inflammation, or any surgical procedures. He is diabetic, and for the last 5 years, he has been on daily 500 mg single dose metformin therapy.

Physical examination revealed a single, well-formed, firm, non-tender, mobile lump of 5 cm × 4 cm × 3 cm on the anterior aspect of the right scrotum. The mass had no connection to the testis, epididymis or spermatic cord. The covering skin was normal with no ulceration. Both testes were normal and no inguinal lymph nodes could be palpated.

Ultrasound scan of the scrotum showed a 40 mm × 20 mm hypoechoic, poorly vascular lesion in the scrotum associated with calcification.

**Fig. 1:**
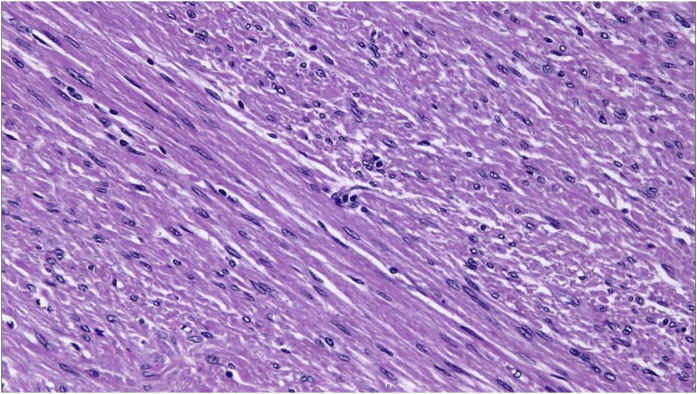
Intersecting fascicles of spindle cells separated by variably collagenized stroma. (H&E stain).

We enucleated the mass under spinal anesthesia and sent the sample for histopathology that showed a benign scrotal leiomyoma that had been completely excised. The cut section demonstrated a well-localized, non-capsulated tumor that was composed of fascicles of spindle cells arranged in intersecting bundles and separated by variably-collagenized stroma. No mitotic figures were seen [Fig. 1].

**Fig. 2:**
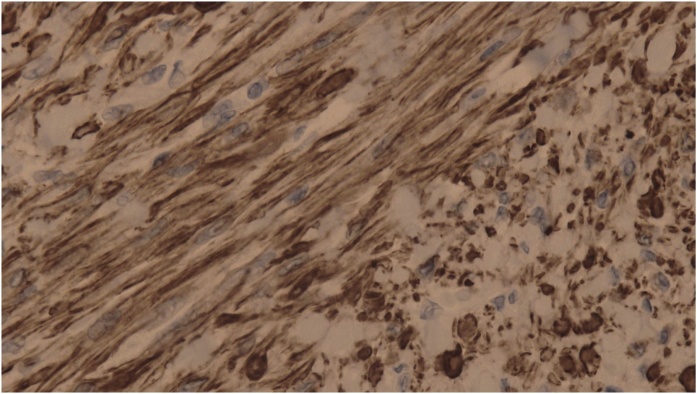
Strong diffuse cytoplasmic staining for desmin in spindle cells.

**Fig. 3:**
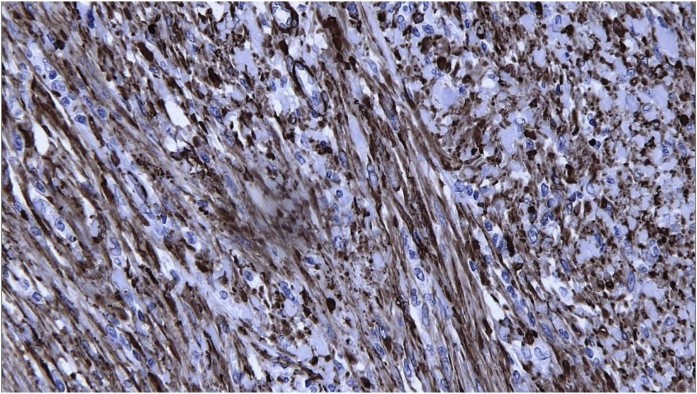
The cells show positive cytoplasmic membrane staining for smooth muscle actin.

Immunohistochemistry was performed with positive control and it revealed a positive reaction to both Desmin [Fig. 2] with diffuse cytoplasmic staining and smooth muscle actin [Fig. 3]. These immunohistochemical findings, together with the physical examination and ultrasound findings, were consistent with a diagnosis of typical scrotal leiomyoma.

## 3 Discussion

Leiomyomas may originate from any location in the genitourinary system where there is smooth muscle [6]. There have been reports of leiomyomas in the renal pelvis, bladder, spermatic cord, prostate, epididymis, and the glans penis [[6], [7], [8]]. It has been suggested that genital leiomyomas have myofibroblastic origins [9]. Among those are scrotal wall tumors. These are usually asymptomatic tumors of the dartos muscle that are commonly seen in Caucasian men [10] and present in the fifth decade of life [11].

Scrotal smooth muscle tumors can be categorized into three. Leiomyomas, atypical or symplastic leiomyomas, which are not hypercellular and lack mitotic activity, and leiomyosarcomas [12]. Pathologically, four features are used to categorize these tumors. They are (i) size more than 5 cm in greatest dimension; (ii) infiltrating margin; (iii) more than 5 mitotic figures per high-power field and (IV) moderate cytological atypia. Benign tumors are those which fulfill only one of the four features. Those fulfilling two of the criteria are atypical or symplastic leiomyomas, while tumors displaying three to four of the criteria are diagnosed as leiomyosarcomas [13].

Most cases reported have been asymptomatic. This leads the patients not to seek treatment until the lesion becomes large and cosmetically undesirable [4]. The largest reported weight is 8 kg [14].

Clinical manifestations of a scrotal leiomyoma may mimic other conditions. Therefore, to correctly identify it, a list of differential diagnoses has to be considered including schwannoma, neurofibroma, dermatofibroma, adnexal tumors, and metastases [4].

Ultrasound is the first-line imaging investigation in patients with suspected scrotal masses. MRI can be more sensitive and accurate but is usually not needed. A definitive diagnosis requires histological examination of a resected specimen [15]. Based on the above-mentioned histological criteria, immunohistochemistry, physical examination, and ultrasound findings, our case was a typical scrotal leiomyoma.

Despite atypical leiomyomas have histological characteristics mimic malignancy, they have a benign course and behave like the typical one even if they have a larger size. Hence both are managed with surgical excision. However follow up is required to detect recurrence. If detected, a thorough investigation should be carried out to rule out any possibility of malignancy. Nevertheless, the use of radiation should be avoided as it may result in malignant transformation. In contrast leiomyosarcomas need a wide 3−5 cm margin resection that includes the subcutaneous tissue and fascia and negative margins [1,16].

## 4 Conclusion

Scrotal leiomyoma is a rare benign mesenchymal tumor of the middle-aged men. The current report describes the clinical and histopathological characteristics to help reduce erroneous diagnoses of this rare tumor, as the method of treatment relies heavily on the correct diagnosis.

## Conflicts of interest

There are no conflicts of interest.

## Source of funding

None to be stated.

## Ethical approval

Approval has been given by Ethical committee of University Of Sulaymanyiah

## Consent

Written informed consent was obtained from the patient for publication of this case report and accompanying images. A copy of the written consent is available for review by the Editor-in-Chief of this journal on request.

## Author contribution

**Operating surgeons:** Saman Salih Fakhralddin and Muhamed Hussen Babarasul

**Design and idea**: Saman Salih Fakhralddin and Rawa Bapir

**Drafting**: Zheen Bahaddin Ibrahim and Muhamed Hussen Babarasul

**Data acquisition**: Muhamed Hussen Babarasul and Rawa Bapir

**Final revision**: Rawa Bapir, Ismaeel Aghaways and Saman Salih Fakhralddin

## Registration of research studies

Not applicable

## Guarantor

The corresponding author is the guarantor of submission.

## Provenance and peer review

Not commissioned, externally peer-reviewed
